# Vocal expression of emotional valence in Przewalski’s horses (*Equus przewalskii*)

**DOI:** 10.1038/s41598-017-09437-1

**Published:** 2017-08-18

**Authors:** Anne-Laure Maigrot, Edna Hillmann, Callista Anne, Elodie F. Briefer

**Affiliations:** 1 Division of Animal Welfare, Veterinary Public Health Institute, Vetsuisse Faculty, University of Bern, Länggassstrasse 120, 3012 Bern, Switzerland; 2 Ethology and Animal Welfare Unit, Institute of Agricultural Sciences, ETH Zürich, Universitätstrasse 2, 8092 Zurich, Switzerland

## Abstract

Vocal expression of emotions has been suggested to be conserved throughout evolution. However, since vocal indicators of emotions have never been compared between closely related species using similar methods, it remains unclear whether this is the case. Here, we investigated vocal indicators of emotional valence (negative versus positive) in Przewalski’s horses, in order to find out if expression of valence is similar between species and notably among Equidae through a comparison with previous results obtained in domestic horse whinnies. We observed Przewalski’s horses in naturally occurring contexts characterised by positive or negative valence. As emotional arousal (bodily activation) can act as a confounding factor in the search for indicators of valence, we controlled for its effect on vocal parameters using a behavioural indicator (movement). We found that positive and negative situations were associated with specific types of calls. Additionally, the acoustic structure of calls differed according to the valence. There were some similarities but also striking differences in expression of valence between Przewalski’s and domestic horses, suggesting that vocal expression of emotional valence, unlike emotional arousal, could be species specific rather than conserved throughout evolution.

## Introduction

Emotions and their expression play an important role in social species. Indeed, perception of emotion expression regulates social interactions by allowing individuals to assess conspecifics’ emotional state and intention of behaviour, and to react in an appropriate manner^1^. Emotions can be characterised by their two main dimensions^2, 3^: valence (negative versus positive; e.g. sad versus happy) and arousal (bodily activation; e.g. calm versus excited). The latter can be considered as the intensity of bipolar valence^4^. The recent application of the dimensional approach to the study of emotions in animals allowed new physiological, behavioural and cognitive indicators of emotions to be highlighted. For example, ear position differs according to emotional valence in sheep (*Ovis aries*)^5^ as well as in cows (*Bos taurus*)^6^, and a low percentage of visible eye white indicates positive emotions of low-arousal in cows^7^. In addition, heart rate and body movement are commonly used as indicators of emotional arousal across species^8–10^. The discovery of clear emotional indicators is crucial for many disciplines, including animal behaviour, neuroscience, psychopharmacology and animal welfare.

Expression of emotions has been suggested to be conserved throughout evolution. Darwin (1872) used his understanding of evolutionary processes to suggest conservation of facial expression by comparing humans and other mammals. Regarding vocal expression of emotions, the existence of common rules governing the structure of vocalisations as a function of the motivation of the producer have been suggested in birds and mammals by Morton^11^. He observed that various species of birds and mammals express their motivation in a similar manner during social interactions; harsh, low-frequency tones tend to be associated with “hostile” social interactions, while high-frequency, pure tones tend to be produced in “fearful”, “appeasing” or “friendly” contexts. These similarities between species suggest that vocal expression of emotions has been conserved throughout evolution. However, since scientific interest in animal emotions only took off in the last 10 to 20 years^12^, it remains unclear whether this is truly the case.

So far, studies mainly focused on vocal indicators of emotional arousal in negative contexts^13^, and the findings suggest that these indicators are fairly similar across species^13, 14^. An increase in the fundamental frequency (“F0”), duration, amplitude, energy distribution, peak frequency, and call rate, as well as a decrease in the inter-call interval has been observed in most species studied to date^13^. For example, with an increase in arousal within negative contexts, call duration increases in pigs (*Sus scrofa*)^15^ and chimpanzees (*Pan troglodytes*)^16^, call rate increases in calves^17^ and silver foxes (*Vulpes vulpes*)^18^, F0 increases in japanese macaques (*Macaca fuscata*)^19^ and rats (*Rattus norvegicus*)^20^, while harmonicity decreases in dogs (*Canis familiaris*)^21^ and bonnet macaques (*Macaca radiate*)^22^. Conversely, vocal indicators of arousal during positive contexts, as well as indicators of valence, have been more rarely investigated^13^. Indeed, finding indicators of valence requires comparing negative and positive contexts of similar emotional arousal. This can be challenging, as positive emotions often trigger lower arousal than negative ones and are, as a result, harder to distinguish from neutral contexts^5^.

Several types of vocalisations have been shown to indicate either positive or negative emotional states. However, few studies have investigated changes in vocal parameters within a given call type as a function of emotional valence (e.g. variation within dog barks – as opposed to between barks and growls, which is equivalent to within human speech – as opposed to between laughter and crying). Yet, such subtle acoustic variations (i.e. that are difficult to perceive by human ear) do occur in calls that are produced in both negative and positive contexts (e.g. rumbles in elephants *Loxondota africana*, barks in dogs, meows in cats *Felis catus*, whinnies in horses *Equus caballus*)^23–26^. These changes could allow conspecifics to perceive the range of emotions expressed by the producer, even within a given context. For example, in elephants, the range of F0 in rumbles decreases from negative to positive valence^23^, while the energy distribution of cat meows increases^24^. In dog barks, call duration decreases and F0 contour increases from negative to positive contexts^25^.

Systematic studies comparing vocal expression of emotions between closely and less closely related species using the same vocal parameters could be very informative regarding the evolution of expression of emotions. In this study, we investigated vocal expression of emotional valence in Przewalski’s horses, with the aim of comparing our findings with domestic horses (*Equus caballus*)^26^. In the wild, where they have been re-introduced since 1992, Przewalski’s horses live in harems (stallion, mares and foals) or in bachelor groups (stallions without a harem)^27^. This species is highly social and relatively vocal. Similarly to the closely related domestic horse, it produces three main types of calls: squeals, nickers and whinnies^28–31^. First analyses of this species’ calls seem to suggest that Przewalski’s stallions’ separation calls tend to be shorter than those produced by domestic stallions, while Przewalski’s mares’ separation calls show a lower frequency than those produced by domestic mares^28, 29^. However, these two studies only focused on nickers and squeals and on a limited sample size and number of acoustic parameters. A thorough examination of Przewalski’s horse vocalisations is thus needed to find vocal indicators of emotions.

In order to find vocal indicators of emotional valence, we compared the types of calls produced by Przewalski’s horses in positive and negative contexts, as well as within-call type changes according to emotional valence. In order to allow a direct comparison with domestic horses, we specifically focussed on the same acoustic parameters as measured in domestic horses in a previous study^26^, including frequencies of the two fundamentals (“F0” and “G0”) identified in domestic horse whinnies. According to the hypothesis of conservation of emotion expression (Darwin 1872), we expected shorter calls as well as a lower G0 (higher fundamental frequency) and F0 (lower fundamental frequency), lower energy distribution (Q25, Q50 and Q75) and higher (AMextent) but slower (AMrate) amplitude modulations in positive compared to negative contexts, similarly to what we found in domestic horse whinnies^26^. Alternatively, expression of emotional valence could be species specific and differ considerably, even between closely related species.

## Results

**Figure 1:**
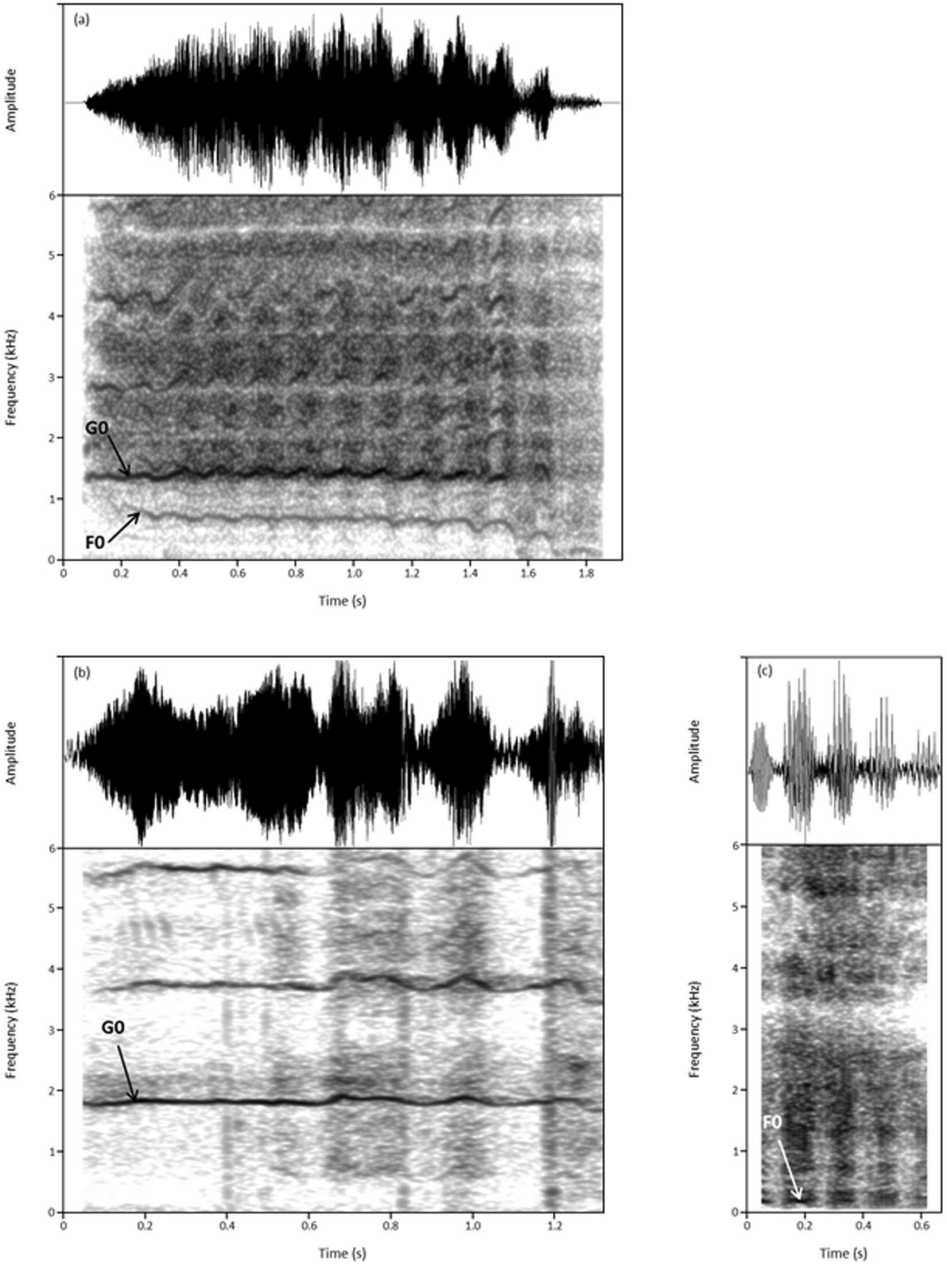
Spectrograms (below) and oscillograms (above) of (**a**) a whinny, (**b**) a squeal and (**c**) a nicker produced by Przewalski**’**s horses. F0 (lower fundamental frequency) and G0 (higher fundamental frequency) are indicated. These calls are available as audio files (Audio S1–S3).

We tested 23 Przewalski’s horses of various ages, living in two different wildlife parks in Switzerland and kept in groups (five different groups comprised of 5 to 14 animals). According to the parks’ policies, we were not allowed to manipulate the animals. Therefore, observations and recordings were made opportunistically during naturally occurring contexts that could be clearly assumed to be of positive and negative valence, using knowledge of the function of emotions and of horse behaviour^32, 33^ (Table 1). These contexts involved anticipation of a food reward and affiliative interactions (assumed positive), as well as agonistic interactions and social separation (assumed negative). We scored the number of whinnies, squeals and nickers produced by the horses in all contexts and measured their acoustic parameters. Because emotional arousal can act as a confounding factor in the search for indicators of valence^13^, we controlled for the effect of this dimension on vocal parameters using body movement, which is a well-established indicator of emotional arousal^9^. This was done by including movement as a control factor (fixed) in the statistical models. This also allowed us to control for potential effects of body movement on breathing, and thus on vocal parameters. The acoustic analysis is described in details in the methods and Supplementary Methods (see Table 2 for definition and abbreviation of the parameters). Przewalski’s horse whinnies were observed to contain, similarly to domestic horses^26^, two fundamental frequencies (F0; lower fundamental frequency and G0; higher fundamental frequency) in every whinny. Nickers and squeals presented only one fundamental frequency, which was similar in frequency to whinny F0 (lower fundamental frequency) for nickers and to whinny G0 (higher fundamental frequency) for squeals (Fig. 1). We thus measured both F0 and G0 in whinnies, F0 in nickers and G0 in squeals, in addition to the following parameters; quartiles of energy, call duration and amplitude modulations (Table 2). We then compared the frequency of occurrence of call types between the two positive contexts and the two negative ones using Chi-square tests, and their acoustic parameters using linear mixed-effects models (LMM). Because we could obtain the body movement scores (indicator of arousal) of the horses only for 72 calls of a total of 194, we present the results of models without the movement included, as well as with the movement included (i.e. after controlling for emotional arousal). Results are presented as residuals of the models controlling for all fixed factors except the factor of interest (e.g. without valence when investigating the difference between negative and positive contexts; which corresponds to the outcome variable after removing the variance linked to the control factors in the model). All means are given with SDs.

**Table 1:** Description of the contexts of production, the behaviours involved and attributed valence^30, 57, 58^.

Context	Description	Valence
Anticipation for a food reward	Caretaker visible to the horses and approaching them with the concentrate and/or a new haystack (maximum 1 minute).	Positive
Affiliative interactions	Interactions that triggered an approach behaviour toward the other horse and a decreased distance between animals (play and allogrooming).	Positive
	*Play: Short event of normal but exagerated behaviours without any clear function, including playful nips, pounces, etc. Unlike in agonistic interactions, the ears are oriented forward or sideways, the lips are protruded, and teeth are covered*.	
	*Allogrooming: Two horses rub each other’s body with mouth and incisors. The ears are held sideways.*	
Agonistic interactions	Interactions that triggered an avoidance behaviour toward the other horse and an increased distance between animals (bite, kick, chase, threat).	Negative
	*Bite: A horse bites another with open mouth. The ears are backwards and the corners of the mouth pulled back.*	
	*Kick: A horse strikes backwards toward another with a hind leg and the ears are backwards.*	
	*Chase: A horse pursues another with ears backwards.*	
	*Threat: A horse threats another to kick or bite by showing the same behaviours.*	
Social separation	Separation of the group in two parts. Half of the initial group was lead to another enclosure out of view from the others.	Negative

**Table 2:** Definition of the types of calls, and of the vocal parameter measured, along with their abbreviations^26, 30, 38^.

	Abbreviation	Description
Call types	Whinny	Longest, loudest and most common calls, which begin with a squeal-like structure and end with a nicker-like one
	Squeal	Loud calls with a high pitch and few amplitude modulations
	Nicker	Low-pitch calls that are short and gutturally pulsated
Acoustic parameters	Duration (s)	Total duration of the call
	G0mean (Hz)	Mean G0 frequency value across the call
	G0range (Hz)	Difference between the maximum and minimum G0 frequency values measured across the call
	F0mean (Hz)	Mean F0 frequency value across the call
	F0range (Hz)	Difference between the maximum and minimum F0 frequency values measured across the call
	TimeMaxF0 (%)	Percentage of the time when F0 is at the maximum frequency value
	AMrate (s-1)	Number of complete cycles of amplitude modulation per second
	AMextent (dB)	Mean peak-to-peak variation of each amplitude modulation
	Q25 (Hz)	Frequency value at the upper limit of the first quartile of energy
	Q50 (Hz)	Frequency value at the upper limit of the second quartile of energy
	Q75 (Hz)	Frequency value at the upper limit of the third quartile of energy

### In which contexts are squeals, nickers and whinnies produced?

More whinnies (*χ* ^2^ = 15.75, df = 1, *p* < 0.0001), and more squeals (*χ* ^2^ = 69.18, df = 1, *p* < 0.0001) were produced in negative contexts (social separation, agonistic interactions) than in positive ones (anticipation for food, affiliative interactions; Table 3). By contrast, more nickers occurred in positive than in negative contexts (*χ* ^2^ = 5.45, df = 1, *p* = 0.012; Table 3). In addition, within each valence, we found that squeals were produced more often during agonistic interactions compared to social separation (negative valence, *χ* ^2^ = 81.39, df = 1, *p* < 0.0001), and during anticipation for a food reward, compared to affiliative interactions (positive valence, *χ* ^*2*^ = 5.44, df = 1, *p* = 0.020; Table 3). The distribution did not differ between contexts within each valence for whinnies and nickers (*p* ≥ 0.07 for all; Table 3).

**Table 3:** Number of whinnies, nickers and squeals analysed for each valence, each context and for each horse (including range; n = 23 horses).

Valence	Context	Whinny	Nicker	Squeal
Negative	Total	46	9	93
	Separation	29	5	3
	Agonistic interaction	17	4	90
Positive	Total	15	22	9
	Food reward	11	7	8
	Affiliative interaction	4	15	1
**Total**		**61**	**31**	**102**
**Number per horse (mean ± SD)**		**4.1 ± 3.9**	**2.8 ± 3.8**	**6.4 ± 6.8**
**Range per horse**		**0 to 15**	**0 to 14**	**0 to 30**

### How are calls modulated according to emotional valence?

Our analysis of vocal parameters as a function of the putative emotion triggered by naturally occurring contexts revealed one parameter that was influenced by emotional valence. Q75 (LMM: F_1,62_ = 4.71, p = 0.034) decreased from negative (mean ± SD model residuals: 28.2 ± 582.5) to positive (−90.7 ± 525.4) valence, across call types. All other parameters were not significantly affected by the putative emotional valence (*p* ≥ 0.13 for all).

Adding the movement level (0–3; indicating the emotional arousal and/or effect of breathing) in each model revealed that six parameters were influenced by the valence of the emotion after controlling for the variance explained by the movement (see Table 4 for mean ± SD and statistical results). G0range, as well as AMrate, Q50 and Q75 (energy quartiles) all decreased from negative to positive valence, while AMextent and TimeMaxF0 increased (Table 4). To summarize, calls produced in positive contexts were lower in frequencies (energy quartiles), with a more stable G0 (higher fundamental frequency; G0range), and with larger (AMextent) but less (AMrate) amplitude modulations, while the lower fundamental frequency (F0) peaked later in the call (TimeMaxF0).

**Table 4:** Effect of emotional valence on vocal parameters after controlling for emotional arousal (LMM; only significant values are presented).

Parameter	NumDF	DenDF	*F*	*P*	Valence	Mean	SD	Variation
G0range	1	35.12	6.26	**0.017**	Neg	**0.07**	1.42	>
					Pos	−**1.31**	1.23	
AMrate	1	52.18	8.31	**0.006**	Neg	**0.16**	2.97	>
					Pos	−**0.73**	3.21	
AMextent	1	46.76	7.26	**0.010**	Neg	−**0.01**	0.23	**<**
					Pos	**0.07**	0.42	
Q50	1	61.41	5.35	**0.024**	Neg	**26.07**	459.96	>
					Pos	−**118.33**	333.31	
Q75	1	59.12	10.70	**0.002**	Neg	**42.84**	556.07	>
					Pos	−**194.43**	520.73	
TimeMaxF0	1	26.00	5.80	**0.023**	Neg	−**0.18**	0.76	**<**
					Pos	**0.21**	1.24	

The mean ± SD residuals of the models controlled for sex, age, size of the group, type of call and body movements are indicated (see Table S1 for raw values). The direction of the effect is indicated (‘<’ indicates an increase from negative to positive valence, whereas ‘>’ indicates a decrease).

### How do call types differ?

Our analyses of the effect of the type of call on the vocal parameters revealed seven parameters that differed according to this factor (see Table 5 for mean ± SD and statistical results). Overall, the amplitude modulation rate (AMrate) was lowest in whinnies and highest in squeals. The parameters related to the energy quartiles (Q50 and Q75) were lowest in nickers and highest in whinnies, which were also the longest calls, while squeals were the shortest ones (Duration). The range of G0 (higher fundamental frequency; G0range) was higher in whinnies than in squeals (not present in nickers). In addition, the parameters related to F0 (lower fundamental frequency; F0mean and F0range; not present in squeals) were both lower in nickers than in whinnies. All the remaining parameters were not significantly affected by call type (*p* ≥ 0.26 for both; Table 5). To summarize, whinnies were the longest vocalisations. They were higher in frequency than squeals (energy quartiles) and nickers (energy quartiles and F0mean). They also had a larger range of G0 (higher fundamental frequency; G0range) than squeals and a larger range of F0 (lower fundamental frequency; F0range) than nickers. Squeals, on the other hand, had more amplitude modulations (AMrate) than both nickers and whinnies.

**Table 5:** Effect of the type of call on vocal parameters (only significant values are presented).

Parameter	NumDF	DenDF	*F*	*P*	Call type	Mean	SD
Duration	2	96.09	29.08	<**0.0001**	Whinny	**0.11**	0.10
					Nicker	−**0.05**	0.18
					Squeal	−**0.05**	0.20
G0range	1	130.95	21.32	<**0.0001**	Whinny	**0.43**	1.07
					Nicker	−	—
					Squeal	−**0.26**	1.42
F0mean	1	69.39	9.72	**0.003**	Whinny	**13.28**	87.38
					Nicker	−**25.71**	36.51
					Squeal	—	—
F0range	1	90.72	10.88	**0.001**	Whinny	**0.17**	0.90
					Nicker	**−0.32**	0.85
					Squeal	—	—
Amrate	2	106.55	226.18	<**0.0001**	Whinny	**−3.98**	3.80
					Nicker	**−2.13**	4.17
					Squeal	**3.03**	5.58
Q50	2	171.62	34.10	<**0.0001**	Whinny	**238.65**	449.33
					Nicker	**−347.55**	312.99
					Squeal	**−37.10**	510.55
Q75	2	151.68	33.16	<**0.0001**	Whinny	**173.11**	413.61
					Nicker	**−492.72**	504.51
					Squeal	**46.22**	640.55

The mean ± SD residuals of the models controlled for sex, age, size of the group and emotional valence are indicated (see Table S2 for raw values).

### Do vocal indicators of valence differ between call types?

The analyses of the interaction effect between the putative emotional valence of the context and the type of call produced revealed two parameters (AMextent and Q25), for which valence-related changes depended on the call type. AMextent increased from negative to positive valence in nickers (mean ± SD model residuals: negative, −0.09 ± 0.43; positive, 0.04 ± 0.33) and squeals (negative, −0.002 ± 0.23; positive, 0.02 ± 0.28), while it decreased in whinnies (negative, 0.02 ± 0.35; positive, −0.06 ± 0.40; interaction effect: *F*_2,193_ =3.28, *p* = 0.040). On the other hand, Q25 increased in nickers (negative, −0.03 ± 0.18; positive, 0.01 ± 0.28) and decreased both in squeals (negative, 0.003 ± 0.65; positive, −0.03 ± 0.96) and in whinnies (negative, 0.03 ± 0.83; positive, −0.11 ± 1.20; interaction effect: *F*_2,192_ = 3.91, *p* = 0.022).

Adding the movement level (0–3; indicating the emotional arousal and/or effect of breathing) in each model did not reveal any parameter that was significantly influenced by the interaction between call type and valence (*p* ≥ 0.09 for all). To summarize, before controlling for emotional arousal (reduced sample size), the direction of changes in the extent of amplitude modulations (AMextent) and in the lowest energy quartile (Q25) as a function of the emotional valence varied depending on the call type.

## Discussion

In this study, we investigated whether the type of call produced, and the acoustic structure of vocalisations, changed between contexts characterised by different emotional valence, in order to identify vocal indicators of emotional valence in Przewalski’s horse. We predicted that, if emotion expression has been conserved throughout evolution (Darwin 1872), the indicators of emotional valence revealed in our study would be similar to those revealed in closely related species (domestic horse^26^), as well as in other, more distant species^13^. We found that nickers were more often produced during positive contexts (anticipation of a food reward and affiliative interactions), while whinnies and squeals were occurring more often during negative contexts (agonistic interactions and social separation). In addition, squeals were especially produced during agonistic interactions compared to social separation. Further, we found that the acoustic structure of calls differed according to the valence of the contexts and that some of these changes were call-type specific. These between- as well as within-call type differences between negative and positive contexts could help the animals to foresee conspecific’s reactions, hence enabling them to better cope with social interactions. The perception of these vocal indicators by conspecifics could eventually lead to emotional contagion, which is the first level of empathy^34^.

We found that the type of calls produced differed according to the valence of the contexts. It thus seems that the type of call gives important information about the emotional valence experienced by the animal. In addition, after controlling for variation linked to emotional arousal and/or breathing (using body movement as an indicator), parameters describing G0 range, as well as energy quartiles (Q50 and Q75) and the number of amplitude modulations (AMrate) decreased across call types from negative to positive valence. By contrast, the extent of amplitude modulations (AMextent) and the percentage of the time when F0 was at its highest value (TimeMaxF0) increased. The production process of G0 in horses is not known yet. Therefore, we do not know if F0 and G0 are produced through the same mechanism. Yet, a decrease in F0range from negative to positive valence has been found in goats^8^ and elephants^23^. Similarly, lower variation in F0 in positive contexts than in negative ones has been observed in dog barks^25^. This suggests that a decrease in the fundamental frequency range (G0, higher fundamental frequency in horses or F0, lower fundamental frequency in other species) could be an indicator of valence that has been conserved throughout evolution. In addition, the energy distribution decreases between negative and positive contexts in squirrel monkeys (*Saimiri sciureus*)^35^, similarly as what we found in Przewalski’s horses, but increases in cats^24^ and in pigs^36^. Those parameters thus seem to change in an inconsistent manner across species, indicating that they might be more species specific.

To investigate how vocal expression of emotions evolved, we specifically compared our results to vocal indicators of emotional valence highlighted previously in domestic horse whinnies^26^. Domestic and Przewalski’s horses are closely related. Even though Przewalski’s horses are not the ancestors of domestic horses, they remain the closest non-domesticated relatives of domestic horses. Indeed, the two lineages (Tarpan horses; *Equus ferus ferus* and Przewalski’s horses; *Equus ferus przewalskii*) diverged ~45,000 years ago, before horse domestication, which occurred ~5000 years ago^37^. In Przewalski’s horses, as revealed in this study, whinnies had lower energy distribution (Q25, Q50 and Q75; Q25 decreased in whinnies before controlling for movement and was not significant afterwards), slower (AMrate) and lower (AMextent; decreased specifically in whinnies before controlling for movement and increased across call types afterwards) amplitude modulations, and narrower G0range in positive compared to negative contexts, while they had higher TimeMaxF0. Some of these changes are similar in domestic horses^26^. In this species, whinnies were shorter, and showed lower G0mean, lower energy distribution (Q25, Q50 and Q75) and slower (AMrate, marginally significant) but higher (AMextent) amplitude modulations in positive than in negative contexts. Therefore, a decrease in energy quartiles and AMrate from negative to positive contexts are changes shared between the two species. In addition, we observed that Przewalski’s horse whinnies all show two fundamental frequencies (F0; lower fundamental frequency and G0; higher fundamental frequency), suggesting biphonation, similar to domestic horses^26^. However, whinny duration and the frequency of G0, which were identified as the most reliable indicators of valence in domestic horses^26^, did not vary as a function of the valence of the contexts in Przewalski’s horse whinnies. The dissimilarities observed between these two species could be due to genetic differences or could have arisen during the domestication process, since selection pressures acting on vocal communication largely differ between wild (e.g. presence of predators, open environment) and domestic settings (e.g. interaction with humans, restricted and protected environment). Why biphonation has been maintained across both species of Equidae is a subject for further research, which could shed light on the evolution of acoustic communication in Equidae.

Interestingly, some parameters varied differently with the valence according to the type of calls. Before controlling for emotional arousal, AMextent and Q25 both increased from negative to positive valence in nickers, both decreased in whinnies, while in squeals, AMextent increased and Q25 decreased. The opposite pattern observed in nickers and whinnies could be due to the different functions of these calls. Indeed, nickers are mainly produced as anticipatory calls, contact calls and to indicate sexual attraction, and their acoustic structure is adapted to short-range communication^38^. By contrast, whinnies are produced in a larger variety of situations. They  are especially used as separation calls and to maintain social contact at a distance and thus need to be heard from far away^30^. These two call types (nickers and whinnies) are thus produced under different environmental and social constraints. Additionally, nickers are composed of one fundamental frequency (F0), while whinnies are composed of two fundamental frequencies (F0 and G0). This suggests that these two call types may not be produced through the same vocal/anatomical mechanism, which could contribute to these differences. Further research into horse vocal production is required to understand how these calls are produced. Similar differences between call types have been found in piglets in relation to emotional arousal^39^. It has been suggested that these differences between call types in the way arousal and valence are encoded could explain dissimilarities observed between- and within-species in vocal expression of emotions^39^. Overall, these results indicate that the way emotions are encoded in vocalisations may vary according to the structure and/or the function of call types and that the specific call type investigated should be accounted for when interpreting results.

As it has been previously suggested^30, 31^, we found several differences between the tree types of calls that we analysed. Indeed, compared to whinnies, squeals were generally shorter, with lower G0range and higher AMrate as well as lower energy quartiles (Q50 and Q75). Nickers, on the other hand, were also shorter than whinnies, and showed a higher AMrate (lower than in squeals though), as well as lower frequencies (Q50, Q75, F0mean) and lower F0range. Squeals (high in frequency and relatively pure tone) are produced during agonistic contexts, painful contexts or sexual encounters. Nickers (low in frequency and relatively harsh) are produced just prior to feeding, as greeting calls, when a stallion expresses his sexual interest in a mare or when a mare calls her foal and encourages it to move closer to her. Finally, whinnies (begin with a squeal-like structure and ends with a nicker-like one) are produced in a variety of contexts^30, 31^. These results do not conform to Morton’s “motivation-structural rule”^11^, which suggests that animals often produce low-frequency calls in hostile contexts and high tonal calls in fearful, appeasing or friendly contexts. Nevertheless, squeals can sometimes be described as calls indicating fear or attempt to appease, as they are often produced by the victims of an aggression or a sexual encounter^29^. As highlighted by August and Anderson^40^, friendliness and fear represent very different motivational states, and calls produced in these contexts seem to present considerable variation. Therefore, while squeals, as fearful or appeasing vocalisations, might comply with the motivation-structural rule, nickers could be more similar to the low-frequency and noisy sounds described in Carnivora (“purr”^41^).

In this study, we used body movement to control for the effect of emotional arousal on vocal indicators. Indeed, this behavioural parameter has been shown to be correlated to emotional arousal in several species, such as goats and domestic horses^8–10, 26^. Including movement as a control factor also allowed us to control for the effect of gait, and thus breathing, on the vocalisations. However, further tests using physiological measures of arousal instead of behavioural indicators would be useful to verify our findings.

## Conclusions

By using recent advances to measure animal vocalisations and emotions, we found some similarities but also striking differences in the way Przewalski’s horses vocally express their emotional valence compared to other species, including the closely related domestic horse. Indeed, although AMrate and the energy quartiles (Q50 and Q75) varied in the same direction in the whinnies produced by both Przewalski’s and domestic horses, whinny duration and the frequency of G0, which were identified as the most reliable indicators of valence in domestic horses, were not affected by emotional valence in Przewalski’s horses. Although studies investigating vocal expression of emotional valence are still sparse, these results suggest that vocal expression of emotional valence, unlike vocal expression of emotional arousal^13, 14^, could be species specific rather than conserved throughout evolution. Further studies investigating the perception of the indicators that we highlighted in Przewalski’s horses by conspecifics and/or heterospecifics could give us a better understanding of their evolution. These non-invasive vocal indicators would be particularly useful for assessing Przewalski’s horse emotions, since most members of this endangered species live in parks and cannot be easily manipulated.

## Methods

### Subjects

The study was conducted between May and June 2014 in two Wildlife Parks in Switzerland (Wildpark Bruderhaus and Wildnispark Zürich Langenberg), on 23 Przewalski’s horses (17 females and 6 stallions). Seventeen horses were adults (more than three years old) and six were yearlings (less than two years old). They were housed in paddocks (from 70 to 150 m^2^) with access to an adjacent field. They had all been in their group for at least one year. Routine care of the animals was provided by the park employees. The animals were fed twice per day with commercial concentrate (around 9:00 am and 3:00 pm) and they had ad libitum access to hay (and/or grass).

### Observations

Each group was observed for as many days as there were individuals in the group (e.g. 6 days of observation for a group of 6 horses), during 3 h per day. We conducted half of the observations around the morning feeding hours (from 7:30 am to 10:30 am), and the other half around the afternoon feeding hours (from 3:00 pm to 6:00 pm).

### Determination of the emotional valence of the contexts

The following contexts were observed; anticipation for a food reward, affiliative interactions, agonistic interactions and social separation (see Table 1 for description). In the absence of well-established behavioural indicators in Przewalski’s horses, the valence experienced by the horses during vocal production was inferred from the context while considering the function of emotions and equine behaviour^8, 26^. Positive emotions result from encounters with rewarding stimuli that enhance fitness, and trigger approach behaviour towards the reward. By contrast, negative emotions result from encounters with punishing stimuli that threaten fitness, and they result in avoidance behaviour^2^. Accordingly, we considered anticipation for food and affiliative interactions as positive contexts^5, 42–45^. By contrast, social separation and agonistic interactions were considered as negative contexts^42, 43, 46^. For the two types of interactions (affiliative and agonistic interactions), we analysed the calls produced from the moment one animal was approaching another (or for 10 s before the interaction if the approach took longer) until 10 s after the interaction ended.

### Determination of the emotional arousal of the contexts

In order to control for the effect of emotional arousal on horse vocalisations, since it can be a confounding factor in the determination of indicators of valence, we measured body movements, which has been highlighted as a reliable arousal indicator in several species^9^, including domestic horses^26^.

### Data collected

Calls were recorded from outside the enclosures, at distances of 5 to 30 m from the vocalising animal with a Sennheiser MKH 70 directional microphone, connected to a Marantz PMD 661 MK II digital recorder (sampling rate = 44.1 kHz). Accurate individual identification was performed using individual characteristics of the horses (e.g. body size, head size, coat markings, mane length). Recorded vocalisations were uploaded to a computer and saved in a WAV format at 16-bit amplitude resolution. We used Praat v.3.61 DSP Package^47^ for the acoustic analyses. Calls were individually visualised on spectrograms in Praat (FFT method, window length = 0.01 s, time steps = 1000, frequency steps = 250, Gaussian window shape, dynamic range = 60 dB). Vocalisations with high levels of background noise (as visualised on the spectrogram) were not considered for acoustic analysis. Additionally, we filmed the contexts where horses were vocalising whenever possible (when the camera was oriented towards the individuals that were interacting), using a Canon Legria FS2000 camcorder.

### Data analysis

Calls were classified as nicker, squeal or whinny according to their acoustic characteristics (Table 2; Fig. 1; Audio S1–S3 ^30, 38, 48^). We analysed all good quality calls (Table 3) that were separated by at least 10 s intervals, in order to prevent pseudoreplication (consecutive calls are more likely to be homogeneous). Both the call-type classification and the analysis of the acoustic structure was carried out while blind to the contexts (hence valence) of production.

We extracted all vocal parameters using a custom built program in Praat^49^. This program batch processed the analyses and the exporting of output data. In order to prevent biases linked to the settings used for the analyses, the same settings were used to analyse all the calls of a given subject. In total, we analysed 11 parameters, which have been shown to vary as a function of emotions in other species^13^. Source-related vocal parameters were measured by extracting the fundamental frequency contour of each call. It was recently shown that domestic horse whinnies are composed of two fundamental frequencies that are not harmonically related, “F0” and “G0”, suggesting biphonation. Since these two frequencies vary as a function of the emotion of the producer in domestic horses^26^, and since Przewalski’s horse whinnies presented a similar structure, we decided to analyse their contour in the present study. We extracted the higher fundamental frequency (G0) after high-pass filtering whinnies above the lower fundamental frequency (F0; 600–1400 Hz) to isolate G0. Similarly, we extracted F0 after low-pass filtering the calls at 200 Hz above this fundamental frequency (F0; 800–1600 Hz)^26, 50^. Nickers and squeals presented only one fundamental frequency, which was similar in frequency to whinny F0 for nickers and to whinny G0 for squeals. Therefore, we used the same settings as for whinnies’ F0 to analyse the fundamental frequency contour of nickers, and the same settings as for whinnies’ G0 to analyse the fundamental frequency contour of squeals. From F0 and G0 contour, we extracted five parameters that could be measured in whinnies (Table 2; G0mean-TimeMaxF0), three parameters that could be measured in nickers (Table 2; F0mean-TimeMaxF0), and two parameters that could be measured in squeals (Table 2; G0mean and G0range). Additionally, we included in our analyses the frequency values at the upper limit of the first (Q25), second (Q50) and third (Q75) quartiles of energy^51–53^. We measured intensity characteristics by extracting the intensity contour of each call and included in our analyses the number of complete cycles of amplitude modulation per second (AMrate), the mean peak-to-peak variation of each amplitude modulation^54^ (AMextent) and the total duration of each call (Duration). The vocal parameters measured are listed in Table 2 and more details (e.g. settings) are given in the Supplementary Methods.

The body movements were scored from the videos of the contexts in which calls were produced, while blind to the valence attributed to the contexts, but not to the contexts themselves, which were visible on the videos. They were scored using Interact software v. 9.0.7 (Mangold International GmbH, Arnstorf, Germany) for 15 s before each call. We then attributed a score to each gait; 0 for standing, 1 for walking, 2 for trotting and 3 for cantering. These scores were assumed to reflect different levels of emotional arousal for the analyses. Because we were not able to film all instances of call production, these arousal scores were available for 72/194 calls (n = 23 individuals).

### Statistical analysis

We carried out linear mixed-effects models (LMMs) to test the effect of emotional valence on the vocal parameters measured. Statistical analyses were performed in R (version 3.3.1, R Development Core Team, 2015) using the lmer function from the lme4 package^55^. The models included the vocal parameters as a response variable (one model per parameter) and the sex (female or male) and age (yearling or adult) of the horses, as well as the size of the group (5 to 13 individuals) as fixed factors to control for their effects. The type of call (whinny, nicker or squeal), the valence of the context (positive or negative) and the interaction between these two parameters were also included as fixed factors. Finally, we included the context (anticipation for food, affiliative interactions, social separation and agonistic interactions) nested within the identity of the horses, itself nested within the group as a random factor crossed with the date of the observation. This allowed us to control for repeated measurements of the same context and subject, and for differences between groups (e.g. distance to the microphone, which was specific to each group) and days of recording. Non-significant interactions between the type of call and the emotional valence were removed from the final models^56^.

In order to control for the effect of body movement (and thus of arousal and/or differences in breathing linked to body movement) on vocal parameters, we ran, on all the parameters, a second series of models including body movements (scores corresponding to the subject’s gait: 0–3) as an additional fixed factor (continuous). This allowed us to control for this factor without considerably reducing our sample size from the beginning, as body movement was available for only 72/194 calls.

For all models, we checked the residuals graphically for normal distribution and homoscedasticity. To satisfy assumptions, we used a log transformation for G0mean, TimeMaxF0, F0range, AMextent and Q25 (see Table 2 for abbreviations). Some parameters were also cube root transformed (G0range and Duration). These log and cube root transformed vocal parameters were then entered into models fitted with Gaussian family distribution and identity link function. *P*-values were calculated based on Satterthwaite’s approximations (anova function, lmerTest package in R). All models were fitted with restricted maximum likelihood (REML) estimation. In addition, we performed Chi-square tests to compare if the distribution of the different call types within each valence and each context was significantly different from a set of expected values. The significance level was set at α = 0.05.

### Ethical note

Animal care and all experimental procedures were carried out in accordance with the guidelines for the treatment of animals in behavioural research and teaching of the Association for the Study of Animal Behaviour (ASAB, 2012) and the current laws of Switzerland. Wildpark Bruderhaus and Wildnispark Zürich Langenberg are open to the public and the Przewalski’s horses are habituated to the presence of people. This allowed us to approach the horses close enough to conduct observation from the outside of the enclosures. During the recordings, the animals were never manipulated or isolated for the purpose of our study﻿. Observations were carried out opportunistically when horses were spontaneously interacting and fed or when they were separated (in different fields) by the parks authorities.

## Electronic supplementary material

Supplementary Methods and Tables

Audio S1

Audio S2

Audio S3
