# Supplementary material for: Vocal expression of emotional valence in Przewalski’s horses (Equus przewalskii)
Source: Sci Rep. 2017 Aug 18;7:8779. doi: 10.1038/s41598-017-09437-1 (PMC5562828; doi:10.1038/s41598-017-09437-1)
Supplement: Supplementary file 1 — Supplementary Methods and Tables [file 41598_2017_9437_MOESM1_ESM.doc]

**Vocal expression of emotional valence in Przewalski’s horses (*Equus przewalskii*)**

Anne-Laure Maigrot^1, 2^, Edna Hillmann^2^, Callista Anne^2^, Elodie F. Briefer^2^

^1^ Division of Animal Welfare, Veterinary Public Health Institute, Vetsuisse Faculty, University of Bern, Länggassstrasse 120, 3012 Bern, Switzerland

^2^ Institute of Agricultural Sciences, ETH Zürich, Universitätstrasse 2, 8092 Zürich, Switzerland

**Corresponding authors:**

Anne-Laure Maigrot; Tel: +41 (0)44 633 84 38; Address: Institute of Agricultural Sciences, ETH Zürich, Universitätstrasse 2, 8092 Zürich, Switzerland

Elodie Mandel-Briefer; Tel: +41 (0) 44 632 84 55; Address: Institute of Agricultural Sciences, ETH Zürich, Universitätstrasse 2, 8092 Zürich, Switzerland

**Supplementary Methods: Acoustic analysis**

In this section we provide an additional description of the acoustic analysis. The source-related acoustic features and intensity features that we measured (11 parameters) are detailed below (Praat commands are indicated in brackets). We extracted all vocal parameters using a custom built program in Praat, which batch processed the analyses and the exporting of output data^1^.

Przewalski’s horse’s whinnies, as domestic horse’s ones present two fundamental frequencies; “F0” (range = 34-790 Hz) and “G0” (range = 683-2340 Hz). We thus conducted our analyses on both fundamental frequencies. To do so, we extracted F0 and G0 contours using a cross-correlation method ([Sound: To Pitch (cc) command], F0: time step = 0.01 s, pitch floor = 30 Hz, pitch ceiling = 500-1000 Hz; G0: time step = 0.01 s, pitch floor = 50 Hz, pitch ceiling = 1300-4000 Hz). Using this method, we were able to follow G0 and F0 throughout most of the calls, after inspection of the detected contour in Praat [Inspect Pitch object command]. Octave jumps were rectified, if necessary, during inspection of the pitch contour detected by the program.

Additionally, we included in our analyses the frequency values at the upper limit of the first (Q25), second (Q50) and third (Q75) quartiles of energy, measured on a linear amplitude spectrum applied to the whole call. These values describe the relative energy distribution in the spectrum. We measured intensity characteristics by extracting the intensity contour of each call [Sound: To Intensity command]. We then included in our analyses the number of complete cycles of amplitude modulation per second (AMrate), and the mean peak-to-peak variation of each amplitude modulation (AMextent)^2^. We also included the total duration of each call (Duration) measured directly on the spectrogram.

Due to the quality of the calls, F0-related parameters could be measured in 60/61 whinnies. All the relevant parameters could be measured in 100% of the nickers and squeals.

**References**

1. Reby, D. & McComb, K. Anatomical constraints generate honesty: acoustic cues to age and weight in the roars of red deer stags. *Anim. Behav.* **65,** 519–530 (2003).

2. Charlton, B. D., Zhihe, Z. & Snyder, R. J. Vocal cues to identity and relatedness in giant pandas (*Ailuropoda melanoleuca*). *J. Acoust. Soc. Am.* **126,** 2721–2732 (2009).

**Supplementary Tables**

Table S1 | Effect of emotional valence on vocal parameters (raw values; only significant values are presented; see Table 4 for statistics).

|  | **Negative** | |  | **Positive** | |
| --- | --- | --- | --- | --- | --- |
| **Parameter** | **Mean** | **SD** |  | **Mean** | **SD** |
| G0range (Hz) | **484.52** | 287.53 |  | **478.93** | 226.32 |
| Amrate (s-1) | **21.58** | 9.85 |  | **14.74** | 6.29 |
| AMextent (dB) | **9.54** | 3.90 |  | **8.11** | 5.17 |
| Q50 (Hz) | **1073.70** | 645.14 |  | **753.24** | 694.34 |
| Q75 (Hz) | **1980.04** | 755.81 |  | **1318.82** | 987.32 |
| TimeMaxF0 (%) | **44.40** | 29.17 |  | **41.12** | 28.85 |

Table S2 | Effect of the type of call on vocal parameters (raw values; only significant values are presented; see Table 5 for statistics).

|  | **Nicker** | |  | **Squeal** | |  | **Whinny** | |
| --- | --- | --- | --- | --- | --- | --- | --- | --- |
| **Parameter** | **Mean** | **SD** |  | **Mean** | **SD** |  | **Mean** | **SD** |
| Duration (s) | **0.74** | 0.44 |  | **0.81** | 0.60 |  | **1.31** | 0.41 |
| G0range (Hz) | **-** | - |  | **424.62** | 277.52 |  | **582.49** | 253.55 |
| F0mean (Hz) | **146.85** | 63.96 |  | **-** | - |  | **291.26** | 200.86 |
| F0range (Hz) | **77.58** | 99.48 |  | **-** | - |  | **221.65** | 202.40 |
| Amrate (s-1) | **12.51** | 4.55 |  | **27.80** | 5.17 |  | **10.63** | 4.24 |
| Q50 (Hz) | **216.23** | 59.46 |  | **984.48** | 609.70 |  | **1416.99** | 565.04 |
| Q75 (Hz) | **598.06** | 423.93 |  | **2017.28** | 767.37 |  | **2121.46** | 612.65 |

Table S3 | Effect of the different control factors included in the statistical models on vocal parameters.

| **Parameter** |  | **Sex** | **Age** | **Group size** | **Body movements** |
| --- | --- | --- | --- | --- | --- |
| Duration | *F-value* | 2.17 | 1.49 | 0.23 | 7.75 |
|  | ***P*** | ***0.19*** | ***0.23*** | ***0.64*** | ***0.008*** |
| G0mean | *F-value* | 0.10 | 0.16 | 1.36 | 1.70 |
|  | ***P*** | ***0.75*** | ***0.69*** | ***0.25*** | ***0.20*** |
| G0range | *F-value* | 3.15 | 3.49 | 0.05 | 7.54 |
|  | ***P*** | ***0.10*** | ***0.067*** | ***0.82*** | ***0.008*** |
| F0mean | *F-value* | 122.89 | 77.51 | 5.20 | 0.51 |
|  | ***P*** | ***0.003*** | ***<0.001*** | ***0.10*** | ***0.48*** |
| F0range | *F-value* | 0.33 | 1.63 | 1.38 | 0.79 |
|  | ***P*** | ***0.59*** | ***0.23*** | ***0.26*** | ***0.38*** |
| TimeMaxF0 | *F-value* | 0.01 | 3.08 | 1.20 | 2.92 |
|  | ***P*** | ***0.94*** | ***0.091*** | ***0.28*** | ***0.099*** |
| AMrate | *F-value* | 1.00 | 1.45 | 0.14 | 3.12 |
|  | ***P*** | ***0.33*** | ***0.24*** | ***0.72*** | ***0.084*** |
| AMextent | *F-value* | 9.18 | 0.01 | 0.12 | 0.62 |
|  | ***P*** | ***0.004*** | ***0.92*** | ***0.74*** | ***0.43*** |
| Q25 | *F-value* | 5.19 | 6.09 | 1.82 | 0.14 |
|  | ***P*** | ***0.034*** | ***0.016*** | ***0.19*** | ***0.71*** |
| Q50 | *F-value* | 1.01 | 5.69 | 1.64 | 0.02 |
|  | ***P*** | ***0.32*** | ***0.020*** | ***0.21*** | ***0.88*** |
| Q75 | *F-value* | 11.56 | 12.53 | 0.75 | 1.05 |
|  | ***P*** | ***0.001*** | ***0.001*** | ***0.39*** | ***0.31*** |

**Supplementary Audio files**

**Audio S1.** Audio file of the whinny shown in Figure 1 (a).

**Audio S2.** Audio file of the squeal shown in Figure 1 (b).

**Audio S3.** Audio file of the nicker shown in Figure 1 (c).
